# *QuickStats*: Percentage* of Adult Workers Aged ≥18 Years Who Reported Being Threatened, Bullied, or Harassed While on the Job,^†^ by Sex — National Health Interview Survey, United States, 2010 and 2015^§^

**DOI:** 10.15585/mmwr.mm6616a8

**Published:** 2017-04-28

**Figure:**
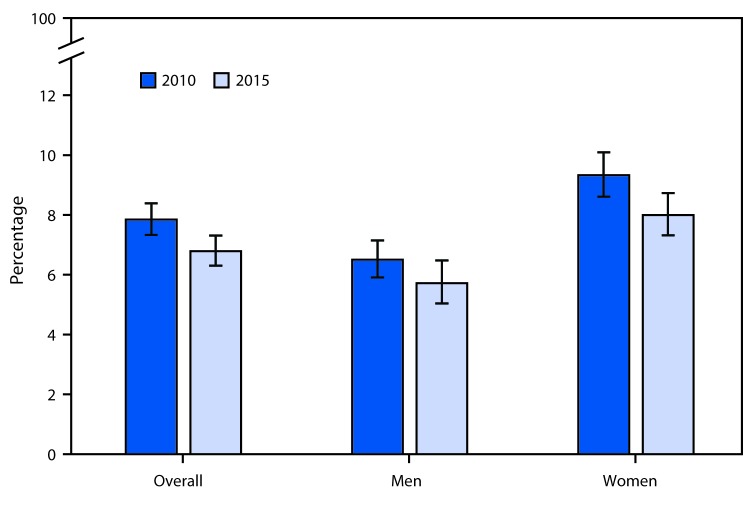
In 2015, 6.8% of adult workers in the United States reported being threatened, bullied, or harassed on the job during the preceding 12 months, down from 7.8% overall in 2010. The percentage of workers who were threatened, bullied, or harassed declined significantly for women but not for men from 2010 to 2015. In both years, women were more likely than men to report being threatened, bullied, or harassed (9.3% compared with 6.5% in 2010 and 8.0% compared with 5.7% in 2015).

**Source:** National Health Interview Survey, 2010 and 2015. https://www.cdc.gov/nchs/nhis.htm.

